# Editorial and Miscellaneous

**Published:** 1861-05

**Authors:** 


### EDITORIAL.

*Practical Suggestion to Writers.*—When you make a prognosis, don't have it printed until the event transpires. You will thus, on the one hand, escape mortification, and, on the other, gain great credit for acuteness and sagacity.

When you operate on a poor devil, always report the case while the patient is still living, or if, as is more than likely, he dies—don't trouble yourself to write out the history beyond the time when he was "as comfortable as could be expected." Remember that the chief point of interest with the profession in cases of this kind is, to know whether the surgeon still survives.

When you treat a fracture, or any case requiring mechanical appliances, never use any apparatus, however well adapted to the case, which any other man has used, or dreamed of using. Thus doing, should your patient accidentally recover with no more than two or four inches of shortening of the limb, or similar deformity, you can report the case with a description of the machinery employed, and thus get some other surgeon prosecuted for malpractice. *Mem.*—Suits of this kind serve to keep the profession before the public, and you perchance may be referred to as authority.

When you write, spare not the adjectives, but "pile up an appalling agony,"—for then shall all readers say: What manner of man is this, that even the "horrible" and "atrocious torments" flee before his scalpel, as consciousness before Letheon vapors!

When you fairly set out to report a questionable case, don't be mealy-mouthed about it—but make a sensation of the raw material, mindful of the (*modern*) scriptural apothegm: "It's of no use to be a fellow, unless you're a devil of a fellow."

When you report any system of treatment adopted by you, it were fitting to record only those cases which escaped with life, especially if after commencing upon the treatment, they present any alarming symptoms. In this manner you can illustrate the wonderful powers of the human system. You see at once if you should publish the cases which died "*in spite of the treatment*," it might tend to prejudice the profession against your system or methods, and *prejudices* ought not to be encouraged in an elevated and enlightened community.

---

*Alcohol in Tuberculosis.*—The Cleveland *Medical Gazette*, commenting on the conclusions of Dr. Davis upon this subject, observes that the author "is an ultraist on this question, and we are satisfied the *present* experience and opinions of the most of the profession do not coincide with these presented by him."

The *American Medical Monthly*, referring to the same paper, says:

We do not know that it would be possible to make Dr. Davis look with complacency upon any use of alcoholic drinks. From his papers, including with this some upon kindred topics, we judge that he is an uncompromising teetotaler; a man very good in his way, but not on that account the most fit person to judge of the effects of alcoholic drinks. Be that as it may, Dr. D. says, that within eight years "the idea was announced and rapidly circulated," that Bourbon whiskey and lager bier would prevent or retard the development of pulmonary tuberculosis. Immediately, he commenced a record of all his well-marked cases of tuberculosis, and this is the result of 210 such cases. Of these, 68 had used alcoholic drinks almost daily; 91 had used them occasionally; 51 had wholly abstained. Of the daily drinkers, only 15 "were such as are usually called drunkards," and 5 of these had delirium tremens when admitted to the hospital.

From these premises the author argues, that these drinks do not prevent or retard the development of tubercle, for these, in fact, are the premises, though drawn out by brief reports of several cases. But the conclusion is not logical. No one has asserted that a person who drinks alcoholic beverages will not have consumption. If such a statement *had* been made, these cases, or rather three-fourths of them, would prove it false. We do not remember to have seen or heard "the idea announced," which was the cause of the statistical record of our author; but in order to meet such a statement, observation must be made on a more extended scale. Thus, it doubtless is true that a good portion of the population of Chicago, especially of the class of poorer foreigners, from whom most of Dr. Davis' patients were drawn, drink wine, beer, or the stronger spirits, either occasionally or habitually. Now, does phthisis pulmonalis prevail among them more than among teetotalers? Do those who are of tuberculous families develop this disease more certainly or more rapidly if they use these beverages than if they totally abstain? We do not intend to be understood to answer these inquiries in the one way or the other, but to show how the author of this paper wanders from the logical answer to the inquiry which is his text.

In fact, Mr. Davis' argument might be turned against his own position, as in this way: Of the adult population of Chicago, it is a large allowance, that *one-tenth never* drink alcoholic drinks, including wines and beer. We find in the record, that of the 210 cases, 51 were teetotalers; that is, nearly one-quarter of the patients were from a class which would be fully represented by one-tenth, 21 instead of 51.

No doubt the records were made and the paper prepared with the best of intentions, but it is our duty to point out the fact that it is illogical, and being so, it fails of its intended purpose as an argument.

May we go so far out of our way as to say that this want of logic has been the cause of many of the errors made by the leaders of the total abstinence movement? Utopian ideas of what should be and can be done, have repeatedly led them to enact laws, such as that known as the Maine law, which have been inevitably followed by a reaction, and intemperance thus been increased, not diminished. But it is always thus with enthusiasts, and we must endure this infirmity of our nature.

It is worth noticing in this connection that there is nothing more worthless than so-called medical statistics, as too frequently compiled. In practical medicine there is scarcely anything more deceitful than the multiplication table. You can accumulate case upon case in myriads, and yet unless there is something "prerogative" about them, they prove nothing.

Is it necessary in this age of progress to repeat this simple philosophical proposition? It would seem so.

Now alcoholic beverages may beget the necessary conditions of tuberculosis, by inducing local changes in the chylopoietic viscera, thus causing imperfection of the nutritive and assimilative processes. So may endemic, epidemic or even sporadic diseases. So may the bran bread and water gruel of the vegetarians. So may debilitating influences of any kind, sexual excesses, mental emotions, inordinate bathing, &c., &c. You can prove each of these propositions by manifold and multitudinous experience and statistics. All these various, and sometimes apparently opposite, causes have one essential link of connection, and this is the grand, Baconian, "prerogative fact" of the series, they prevent or exhaust the nutrient supply of normal blood. Whether we believe that there is excessive metamorphosis of tissue, thus impairing the quality of the blood; or (as some believe) too little metamorphosis, either from deficient sanguineous pabulum or whatever cause, it is seen in every case that we must come back to the blood and blood-influencing processes in every case. It is as true now in the light of modern science, as centuries ago in the light of inspiration: "The Life of man is in the blood." The teeth and salivary glands, the stomach and entire digestive and assimilative organization; a localized organ, or the pervading nervous system; the character of food and drink, the habits or meteorological surroundings, may each, or all combined, tend to render that blood either incapable of building up healthy tissue in a normal manner, or of supplying the waste of metamorphosis—or, perhaps, surcharging it with imperfectly organizable material which exudes from the vessels among the textures, but not to become of them.

An almost infinite variety of remedies have been proposed for "consumption"—and each of them has been from time to time backed up by a formidable array of statistics. Blood-letting and Mercurials, Antimonials and Digitalis, Cimicifuga and Chlorate of Potash, Prussic Acid and Strychnine, Hot

Water and Cold Water, Canada and Cuba, the Mammoth Cave and California, the Cow Stable and the Sugar House, with a multitude other which no man can number even with the aid of the "medical multiplication table," have each had their advocates and their statistics. Legitimate medicine has been sneered at for its apparent contradictions in this respect. The sneer is uncalled for—the scepticism based upon it is unfounded. This is not a Gordian knot which needs cutting even by a surgical scalpel—it can be untied in a trice, by any reasonable thinker. Any—nay, the most antipodal agencies may be required in cases labelled Phthisis.

It is almost incomprehensible that professional gentlemen who have repeated again and again the truism, no case of disease is to be treated simply on its name, but solely according to its indications in the particular case, should now be going about seeking what is not, or is "good for Phthisis." Deluded Friend—Phthisis is not an entity attacking the poor patient as Joab smote Amasa—it is not a spirit from the vasty deep attacking with its unseen javelins the "properties" of the tuberculous lungs, but an effect, the concrete sum of all the causes operating upon the system. Each case is to be scrutinized in all that influences it, and when the morbific causes and results are discovered, they are to be met, not necessarily by Alcohol or even Chlorate of Potash, but, *according to the indications*, precisely as you would rationally treat any other form of disease. We may conceive thus that even the most opposite methods may be called in to assist, not for the phthisis, but to aid in so far as competent in removing its local or general causes.

In the case before us—that Alcohol will beget phthisis is indubitable. Who has not seen cases of "Rum Consumption?" That it will often aid immensely in the relief or actual cure of the disease, are there not abundant evidences? Hundreds of physicians, aye and as good teetotallers as the essayist himself, can attest the fact in their own experience and observatien. "There is a soul of goodness in things evil,"—even in Alcohol. Mercurials have been abused in practice; shall they therefore be abandoned? As elsewhere written, that which is capable of producing a change in the system is truly medicinal and salutiferous, if that particular change is needed. If that change is not needed, it is a poison in use. In this sense Alcohol may become a poison, and, as such, ought not be employed, here or elsewhere.

---

*Syphilization.*—This intolerable, yet apparently interminable, nonsense still finds advocates in Paris. It will be recollected that the first two young men who volunteered inoculation fell victims to the folly. One died of the disease, and the other committed suicide under its deplorable effects upon the system.

M. M. Sperino and Boëck assert:

1. That repeated inoculation with chancrous virus produces immunity.

2. The symptoms of constitutional syphilis disappear under the influence of the inoculation.

3. Syphilization acts in a beneficial manner on the health of the patients.

To all of which we demur and return thereupon the Scotch verdict—*not proven.* It is painful to see such respectable journals as the *Gazette Hebdomadaire* giving publicity to such superlative trash.

It *is not* necessary that because a man is a Doctor, or even a medical writer, that therefore he should be an ass. But if the truth of the propositions above given could be shown, it would be glorious news to some of the Chicago roughs. "A hair of the same dog," &c. *Vive la bagatelle!*

---

*Deaths.*—A very considerable mortality is prevailing among the Medical Journals. We have not space for appropriate obituaries. Not one of them but had a large stock of original vital energy, but *pabulum vitae*, in the shape of paid-up subscriptions, was wanting. *Quis talia fando temperet a —cogitationibus!* Gentle Reader, how stands your account with the C. M. J.?

---

*Philadelphia Colleges.*—It is understood that in consequence of the slight bonds which now hold "these (remaining) United States" together, and in view of the contingency of impending isolation, of that city, all the appointments to positions now, or about to be, vacant, will be made from among the citizens of Philadelphia only. Unpleasant news certainly to sundry outsiders, but clearly politic so far as the residents of that county are concerned. It is always advisable to take in sail during storms.

---

*Ascarides.*—Dr. Wm. Scofield, in the *Journal Mat. Med.*, recommends: "Oil of Turpentine and oil of Pumpkin seed, of each half an ounce; rub them together with the yolk of an egg, and suspend the whole in a pint of water. To be used as an enema." Plain common salt water, or, better perhaps, a suitable quantity of infusion of quassia, or other vegetable bitter, with a spoonful or two of common salt thrown in, is equally as effective and more convenient. We look upon common salt, freely administered, as the best permanent remedy for this sometimes troublesome affection. It is equally efficient against lumbrici. Parents ought to see to it that their children's food is thoroughly seasoned with salt—a thing too often neglected with resulting mischief of this and other sort.

---

*Puerperal Convulsions.*—This subject is again attracting considerable attention from the medical press. Irrational methods based upon false pathology are being discarded in the better circles of practice. It is beginning to be understood that the convulsions of the puerperal state, are by no means analogous to those resulting from apoplexy or encephalic inflammation. Generally the convulsion is a reflex nervous phenomenon—hence amenable to chloroform and other sedatives and antirritants. Sometimes it is really dependent upon uterine hæmorrhage. Convulsions are among the common sequelæ of hæmorrhage whatever the source. They can be produced at will in the lower animals by cutting off or materially reducing the supply of blood to parts. The well known experiments of Dr. Radcliffe forcibly illustrate this. Bloodletting may by its immediate and powerful influence upon the nervous system, sometimes control the eccentric nervous phenomena, but this effect can usually be better secured by other and non-spoliative agents. It is safe to say that excessive bloodletting in puerperal convulsions is not in accordance with the dictates of modern science—nay, more, it is utterly opposed to them. Repeated bloodletting in the same case is either preposterous or worse. Chloroform or Ether, the warm bath, properly selected narcotics, or even stimulants, in their modern use, have wonderfully lessened the terrors and dangers of this formerly often fatal difficulty. Bloodletting possesses a potent influence over the nervous system in common with many other agents; but besides this it wastes and spoils the powers of life. Why use the, collaterally, destructive agent, when others are at hand without attendant peril? The gross mechanical notion of pressure of blood upon the great nervous centres ought by this time to be abandoned, and with it the practice based upon it. Some persons are beginning to ascertain that there is a nervous system, implanted on the organic framework.

---

*Rationale of the Action of Mercurials.*—A contemporary endorses the opinion that the action of Mercurials consists in the *restoration of capillary power*, and considers that this affords "a wonderful clearing up of many of the apparent inconsistencies, incongruities and mysteries of their operation. Their alterative effect in chronic inflammation is immediately revealed. They correct the unhealthy state of the capillaries by causing them to contract upon their contents."

In other words, the restoration or increase; sometimes also, be it recollected, the diminution of secretion; the resolution of tumors and indurations; the absorption of exudations and effusions, and relief of chronic inflammations, is brought about by the capillaries contracting upon and hence squeezing out their contents. Compendious, truly! All that is requisite then to replace this so much dreaded and maligned mineral is to find something which will squeeze out the contents of the capillaries, as he of the "Institutes" has the varied secretions squeezed through *open pores.* Here is a chance for immortality in dethroning King Mercury. Wanted—A power to squeeze out the contents of the capillaries! Will cold, or pressure, or electricity, or animal magnetism do it? Seriously, however, the advance of physiology has shown, if anything, that there must be material changes developed wherever a manifestation of power, or "property," is to be presented. The condition of the capillary depends upon the blood which it contains, the cells and fluids around it, and the nerve conductors by which changes within and around it are influenced. The poisoned, medicated or healthy blood directly impress the capillary parietes; molecular changes at the other extremity of the nerve conductor influence it; direct or communicated modifications in the cells and fluids around control it. Each and all of these are to be taken into account in studying the changes induced in the part affected by this or any other medicine or agent. The contraction of the capillary is but a single incident in the series of phenomena. The microscope is eloquent and conclusive here. The intimate changes characteristic of simple vegetative life are carried on by fluid, capillaries and cells alone—these influenced only by the nutrient fluids drawn from the soil beneath, gases from the air around, and local causes in the growing part. In the animal there is superadded a nervous system which links even the remotest parts, so that a molecular change in one, through the beautiful conducting nerve fibre, influences molecular change and action in the other. Mercury interferes largely with one or all these molecular changes, beneficially or otherwise, as the case may be. It is probable, from the phenomena observed, that it especially increases the rapidity of cell actions, sometimes perhaps diminishing them, but always by changing molecular action in the part. Whether the capillary contracts or dilates thereupon, is altogether a secondary matter, as much so as when a muscle contracts whether extension or flexion results.

How long will, otherwise enlightened, physicians consent to receive either, on the one hand, the flatulent abstractions of old galvanized vitalism, or, on the other, bald mechanical and chemical fancies? It may be laid down as a solid axiom that whatever influences the animal body produces a molecular change in it—thus whether it be from so-called physical or mental forces. It is nonsense to call a man a materialist because he points out the demonstrable fact that even the mind itself, in the spacious or restricted circuit of its musings or manifestations, continually disintegrates the tissue upon which, or through which, it operates. It is this disintegration or molecular change which enables the organized part to manifest its "properties" of whatever character. When we get rid of a little more of the *debris* of old solidism, we shall see more clearly into very many things. The good work is going on, notwithstanding the *reactionaires*, with Don Quixotte-de-la-New-York at their head. Courage, Friends!

---

*Cardiac Disease.*—Prof. Flint, in a recent clinical lecture at New Orleans, observes: "Patients affected with merely functional disorder, have their attention concentrated on the heart, and are with difficulty persuaded that they have not organic disease; while patients really affected with organic disease, are disposed to attribute their symptoms to an affection of some other organ; for example, the liver or the stomach."

This curious fact is attested by other observers. It is well known that in phthisis there is a similar feeling. Pertinent to this is an old Hibernicism by an Irish physician, himself affected with phthisis: "I know, by this token, I have sorra a bit of consoomption about me, for, bedad, I believe I have."

---

*A Practical Treatise on Phthisis Pulmonalis; embracing its Pathology, Causes, Symptoms and Treatment.* By L. M. Lawson, M. D., Prof. of Clin. Med. in the Univ. of Louisiana, &c. Rickey, Mallory & Co., (now Rickey & Carroll,) Cincinnati; 1861. From the Publishers.—"Good wine needs no bush," neither does the name of Prof. Lawson require any introduction to the Northwestern public. Everywhere he is known as an earnest, industrious, acute and sagacious medical scholar and practitioner. When it was first announced that he was about to publish a book on Phthisis, we confess to a temporary dissatisfaction with the choice he had made of a subject. The classic treatise of Dr. Walshe, and the learned, elegant and philosophic work of Prof. Flint, respectively, seemed to leave the field barren for other writers for some considerable time to come. But on perusal of the work as it now lies before us, we admit our mistake, and return our sincerest thanks to Prof. Lawson for his very valuable contribution to American Medical literature.

This is emphatically an American book, and cannot be properly replaced by any other work extant on the subject. The author's investigations upon the subject of the various local climatic influences of our widely extended country, are invaluable and practically highly suggestive. He is of the opinion that the disease is much more prevalent at the North than the South. This opinion, although a very general one, nevertheless has been strongly contested. His argument, from the facts, seem quite conclusive of discussion. The four principal divisions of the subject: Pathology, Etiology, Semeiology and Therapeutics, are each carefully considered and elaborately set forth. Among many things which we should be pleased to reproduce on our pages, we select his summary of the characteristics of tubercle:

"1. The tuberculous element originates in the metamorphosis of the tissues.

"2. It seeks elimination through the lungs, and may continue to pass, in certain quantities, for an indefinite period, without inducing local deposits.

"3. When the morbid element reaches a certain degree of concentration, or when, by long-continued action, it produces a morbid effect on the lungs, local deposits take place.

"4. The first deposit is the elementary morbid substance known as the amorphous stroma; this is followed by the development of molecular granules and peculiar cells, which constitute *tubercle.*

"5. After the existence of solid tubercle for a given period, it softens, and the debris seeks elimination through the bronchial tubes.

"6. The morbid action extends to the adjacent tissues, causes inflammation, softening, and disintegration, too often resulting in fatal disorganization.

"7. The perfect uniformity of tubercle throughout the body, in whatever tissue or organ deposited, exhibits strong evidence of the specific character of the disease, and that it could not originate from the ordinary derangements of nutrition.

"8. The chemical and histological character of tubercle favor the opinion that the whole process is specific in origin and development."

The therapeutics of Phthisis in the main coincide with those adopted by the better informed classes of American physicians. Alcoholic stimulants, tonics, cod liver oil and other nutrients, are the most highly esteemed.

We conclude with his summary of conditions of cure:

"The conditions which justify favorable prognosis may be thus stated:

"1. When the tubercles are limited to one lung, are not very extensive, and have not been associated with inflammation, either as a sequence or an inducing cause.

"2. The general health remaining in a fair condition, without rapid emaciation, fever, or derangement of digestion.

"3. A hereditary tendency to phthisis being slight or entirely absent.

"4. The patient possessing naturally a good constitution, with a sanguineous or nervo-sanguineous temperament.

"5. The occupation being favorable, or at least not of a character to induce phthisis, or the patient being in a condition to make a change to a more suitable business.

"6. The patient having confidence in his medical attendant, and a willingness to submit to treatment, and the ability to avail himself of all incidental means and conditions capable of favoring his recovery, including a change ef climate.

"7. A cheerful and hopeful mental constitution, and a desire to contribute his share to the successful treatment.

"These are conditions which justify a favorable prognosis, although patients may, and often do recover, when the circumstances are much less propitious.

"The unfavorable elements of prognosis may be thus enumerated:

"1. When tubercles occupy both lungs, to a considerable extent, or involve a large portion of one.

"2. When the disease has advanced to the stage of softening, with extensive disorganization of the pulmonory structures.

"3. The general health being greatly impaired, as shown by the existence of extensive emaciation, deranged digestion, hectic fever, and night-sweats.

"4. A decided hereditary tendency to phthisis, and especially if received by a son from a father, or a daughter from a mother.

"5. A naturally feeble constitution, with a phlegmatic or bilious temperament.

"6. The occupation being unfavorable, and the patient not able or willing to make the proper change.

"7. The patient being of a fickle disposition, wanting confidence and perseverance in medical treatment, and unable or unwilling to secure the advantages of a change of climate, and other incidental means of relief."

---

*Tracheotomy in Croup.*—The Boston *Med. Jour.* records two recent cases of tracheotormy in croup, in which each recovered. Ages respectively 5½ yrs. and 1 yr. and 7 months.

---

*Action of Potash, Soda, &c., on the Urine.*—Wm. Moss, M. D., of Philadelphia, reports in the *Am. Jour.*, April 1861, a series of experiments, on himself, with reference to the effects of various substances upon the urinary secretion. As the result of this inquiry, it was found that the Acetate of

Potash still maintains its superiority in every respect as an eliminant through this channel—increasing to the greatest extent both the solid and fluid excretion. Next in efficiency was the Carbonate of Lithia, a saline which has recently been highly commended as an eliminant by Dr. Garrod and others. The latter agent is suggested in cases of gouty diathesis. Thirty grs. render urine the strongly alkaline.

---

*Sydenham and "the rest of Mankind."*—In a recent number of a highly respected contemporary (*Nashville Journal*) are to be found the following observations:

"There is no connection between medicine and state, and politics have never exerted any influence upon the former. It can never become sectional—it never *was* sectional. It is alone bounded by the confines of sin and misery. Its great and good professors lived through the convulsions of revolutions baptized in blood, without considering such political metamorphoses of sufficient importance to speck their immortal pages by a bare allusion. Sydenham wrote and practiced during the troubles of the Cromwellian revolution, but nowhere even incidentally alludes to them. When London, the city of his residence, was almost destroyed by the great fire of 1666, although an event afterwards noticed as exerting a favorable influence upon its diseases, the great master leaves this physical, with the religious and political revolutions of his time, uncommemorated by a word. He, whatever his works as a man, as a physician, lived and moved and had his medical being in a purer atmosphere."

The object of the writer of the above was certainly a most commendable one—particularly at the present disturbed juncture of political affairs. It is fervently to be hoped that medicine may not follow the politicians into the chaos of sectional strife. Nevertheless it is to be hoped that the author does not quote the example of Sydenham, in this respect, as "one worthy of all imitation." On the contrary, it is clear to the professional mind of the present day, that to properly understand man and his diseases, there must be an acquaintance with *all* the influences operating upon him. Not only local causes and the "constitution of the atmosphere," but all those recondite, but immensely potent, causes which impress his nervous system, the greatest disturbing influence to which the material frame responds. The changes of the material atmosphere are slight and trivial in their effect, compared with the tremendous changes wrought, even in man's physical constitution, by the mental and moral influences which surround him. These will vary "the type of disease." They will originate new forms of disease; modify or wholly banish the old. The mental and moral forces, operating through the nervous system of the human being, are not mere fantastic shadows, evanescent and infinitesimal in their impression, but grave deep and lasting marks upon the material frame. The mental and nervous forces are as truly potential in determining molecular changes, and consequent functional manifestations, as are heat and light, electricity and magnetism. It is positively fatal to overlook them—the Hamlet of the play. Sydenham can then only be properly interpreted, when we consider that he wholly overlooked, probably, the dominant influence operating on human bodies. The fault extends even to our times, to our standard books, to our standard methods of practice. Nervous forces are neglected or passed over with slight and scanty notice. We diligently scrutinize the blood and all secreted matters for evidences of change, which sometimes we mistake for the cause of change. We carefully note all meteorological influences, but we too often fail to examine into the highest and strongest forces to which material mechanism succumbs. The forces operating through the nervous system do not exhaust themselves in merely influencing "properties," but in causing positive changes in integral structure, and this not only in nervous and muscular tissue, but upon the blood and all fluids, upon secreting glands and surfaces, and upon all parts where modifications are possible from any cause.

Whilst these influences are capable of causing disease, they are likewise, in the hands of the wise physician, powerful to control morbific action. And this not occasionally or accidentally, but constantly and by well defined laws. We may relieve or cure disease by the changes produced by medicines; we may do the same oftentimes by material changes brought about by the operative energy of the mental and nervous forces. Their laws of action are what now demand the close investigation of all enlightened physicians.

---

*The Pious Dodge.*—Dr. Brannock of Tenn., chronicles in a recent address an instance not unprecedented:

"A young lady who was remarkably nervous and timid was taken sick, and became very much alarmed. To quiet her fears, and the apprehensions of the family, more than from anything serious about the case, a consulting physician was brought in. He came, approached the bed-side, told her not to be alarmed, that he would cure her, called her *daughter*, kissed her, patted her cheeks, smoothed the hair back from her brow, and raising her gently in his arms he gave her a dose of some simple medicine, then kneeling by the bedside he poured out in strains of the most touching eloquence a petition to Almighty God for her recovery. As might be supposed the young doctor was completely laid in the shade; and the sequel to the story was he got no more practice in that family. Dr. so and so was their family physician. He was *such a good man*, they said."

We knew of a case where the consulting physician, in order to convince the patient and family (in a case of common remittent) of his extraordinary zeal in ascertaining the facts of the case, after having gone through with an unusually long and tedious examination, including the use of a stethescope applied to every known part of the body, wound up by dipping his finger in the chamber vessel and tasting the urine! He had him there!

---

*Fracture of the Femur.*—In the N. Y. State Med. Soc. Dr. Swinburne read a paper advocating treatment of fractures of the femur by simple extension. The pelvis is to be fixed by a perineal belt adjusted and secured by means of adhesive straps applied to the leg so arranged as to bring equal pressure upon the integuments and leaving loops through which a cord is passed and fastened to the foot of the bed. No splints or bandages are employed, the powerful muscles and fascia of the thigh answering every indication. He claims very considerable advantages for this method and reports a number of cases during an experience of ten years. Average period of extension five weeks, although in the majority of cases union was firm in three or four weeks. But little extension is needed for the first fifteen days. Extension after that period will secure proper length of the limb.

### AMERICAN MEEDICAL ASSOCIATION.

Why is not something said or done about what every one admits to be not only desirable but an unqualified necessity—the adjournment of the Am. Med. Association until the return of quietness? It is folly—sheer unmitigated folly, to talk about meeting in Annual Session whilst the American body politic is convulsed and the different sections of the country arrayed in arms against each other. It is nonsense to talk about Medicine having nothing to do with politics when the sword is drawn as the arbiter of national destiny, and the avenues of communication between the North and the South are in the hands of irrepressible mobs or armed soldiery with very little respect for the Medical profession ("*per se*") above any other men on their travels. The American Association for the Advancement of Science, long since recognized the stern necessity of the case and prudently postponed their meeting appointed at Nashville. Let those who have the matter in charge do the same thing for Am. Med. Association and thus avoid the inevitable and disgraceful "*fizzle*" which is otherwise as certain as destiny.

Where are the Committee of Arrangements, and why do they not move in the matter at once? Or let the President of the Association promptly issue a manifesto on the subject.

We have waited, for reasons readily suggested to any thinking mind, for those at a distance from Chicago to ventilate the subject—but the stirring events of the hour have evidently caused the Association to be wholly forgotten. It is useless to disguise the matter, the meeting is already adjourned by the condition of the country at large, as effectually as Cromwell adjourned the Long Parliament. Are we to be edified by the pitiable farce of half a dozen ambitious gentlemen assembling in infinitesimal conclave and portioning out the names of the offices among themselves? Is this to be the "lame and impotent conclusion" of the great American Medical Association?

Let us have no idle doubts about constitutional powers to postpone. Whilst you are doubting the thing is done by a higher power—a necessity which knows no law. Rub up your classics, Messieurs Officials, and recollect—*inter arma, leges silent.* We do not wish to be misunderstood—we shall not be misrepresented save by inherently contemptible natures,—we would gladly hail and welcome all our professional brethren, from the Atlantic to the Pacific, from Hudson's Bay to the Isthmus, but we cannot consent to have the Chicago session of the Association hereafter a by-word and a reproach.

Who shall make the official proclamation? We fervently trust that even before this number of our Journal goes out to its readers the words we have written shall have been anticipated in action, either by the President of the Association or by the Committee of Arrangements.

Pray God the time speedily return when the continent shall again see peace, and the peaceful efforts of the Assooiation be nevermore interfered with by the turmoil and confusion of civil war.

### MEETING OF THE ALUMNI OF RUSH MEDICAL COLLEGE.

The invitation to the Alumni of Rush Medical College to meet in this city, the day prior to the time appointed for the session of the Am. Med. Association, we are requested to recall. Now is not the time to commence any such organization as that contemplated in the original call. Events have crowded upon the country which must temporarily put this on one side, as the Association itself is already displaced. Other and better times will come when *alma mater* can greet her alumni under happier auspices. An Association of the Alumni will eventually be formed, but not now when the times are out of joint. When "white-winged Peace" comes again to the distracted country there will be time enough to cultivate all peaceful associations.

---

*Discreditable.*—Scarcely any more striking exhibition of folly and fatuity has recently occurred, than was presented in the present Legislature of Illinois a few days since. The Legislature, at the suggestion of several distinguished medical gentlemen of the State, had provided for a board of Medical Examiners to investigate the respective qualifications of the large number of applicants for surgeoncies in the new regiments. Either to defeat the object of the bill, or to gratify innate littleness, (for which he has been previously somewhat eminent,) a person whom, as a penance for their manifold sins, the people of Chicago had been obliged to elect a member of the Legislature, undertook to foist upon the Board an obscure Homœopathist of this city. It was but a bare chance that the profession escaped the deep disgrace of having this individual elected over the worthily eminent Dr. N. S. Davis, finally chosen. The thanks of the profession are especially due to the Hon. Sol. M. Wilson of Chicago, for baffling the discreditable attempt of the "*wildcat*" legislator.

---

*Surgeons to the Militia.*—It is stated that for the position of surgeons to the new Illinois regiments, (at the time there being but six to be provided for,) there were over *three hundred and fifty* applications. Who after this will undertake to say that patriotism (with good pay,) is at a discount among Doctors? Eyes right! Draw—Scalpel!—Carve—at a venture!

*Pessaries.*—The late work of Dr. Hodge has revived inquiry into the real advantage of pessaries, and other mechanical adaptations, for the support of the uterus in its normal position. Thus far the weight of evidence seems strongly to incline against these agents, as anything more useful than temporary palliatives. The speculum, the pessary, the supporter, *et id omne genus*, have each been prodigiously overrated.

The tendency has been to mistake effects for causes, whether with regard to abrasions, ulcerations, prolapsions or versions.

By far the larger proportion of these various difficulties are directly traceable to causes removable, not so much by medicines or mechanical agencies as by correction of habits and regimen. Thus the abominable load of ' bustle," skirt and petticoat which women will insist upon wearing is chargeable with the greater part. Then the tendency of even the most angelic of the sex to forget the necessity of regular unloading of the reservoirs of excrete matters. Luxurious cushions and (abomination of abominations!) feather beds; want of exercise, and hot, ill-ventilated rooms. A little common sense brought to bear upon the vast majority of these cases will let vastly more light upon them even than peering through a speculum passed (*horribile dictu!*) through a hole in a sheet. If there be any one object we hold in deeper contempt than a "womb doctor," loaded down with his armamentarium, like an ass under heavy panniers, we have yet to discover the luxury of that lower deep.

---

*Dissection Wounds.*—Garrigon revives, in the *Gazette des Hopitaux*, the old practice of keeping the dissection wound soaked in chlorinated water. "This application will always be found efficacious, provided purulent infection have not already set in, when it will be useless."

---

*Sugar Antidotal to Alcohol..*—Pure white sugar is proposed as an efficient remedy for drunkenness. The idea is based upon chemical considerations. It can scarcely prove more than a theoretical medicine in the case, for very obvious reasons. Brandy and sugar notoriously are equally efficient in producing intoxication as "plain brandy—straight."

---

*Asphyxia.*—Prof. Thayer objects to the proposition of "the necessity of keeping up the heat of the body during suspended respiration." His plan in such cases is "to expose the body to the air at a temperature considerably below that of the natural heat of the surface, during the continuance of the efforts for restoring respiration. As soon as respiration is established, the body requires the protection of warm clothing, and in the newly-born especially needs the most thorough covering from the air." He founds his practice upon the results of the experiments of Edwards, Astley Cooper, Nunnely and Marshall Hall. The point is worthy of farther elucidation.

---

*Bloodletting et alii.*—The venerable Dr. Alexander H. Stevens, in some recent remarks before an eastern medical society, is far from occupying the ground which Prof. Gross has so strongly insisted upon. He fully admits the difference in type of diseases at different times and diverse localities. He observes: "Practical men do not treat their cases altogether, or chiefly, according to their place in nosology. Diseases are not mere names. They are not abstractions. Nature knows none such. The condition of the system, more than the name of the disease, guides the skillful practitioner. Disease of the same name sometimes require a purely tonic treatment; at other times a mixed treatment, and again an antiphlogistic treatment throughout all its stages. These variations are those not only of time, but of place."

Formerly having found much benefit from free bloodletting, both in his own case and his patients, he nevertheless says: "I no longer or very rarely find, at the present time, the indications for bleeding as laid down by John Hunter and other observers."

"If the practice of fifty years ago were now put in force, patients would be killed outright. They would die before the physician could get out of the house, and our Galens, instead of being saluted with the words which Dr. Robert Jackson has made familiar, 'Oh, sir, you have cut the throat of the fever,' would be told, 'Sir, you have butchered your patient."

Nevertheless it is not fitting or right to say that the practitioners of old time were much in error—simply because it is clear that the type of disease has changed, and we may expect will continue to change, as men and their surroundings undergo various mutations. But in one thing there will be no mutation; so long as there remains respect in the human heart for *the true physician*, so long will Alexander H. Stevens, the Nestor of the American Profession, remain *clarum et venerabile nomen.*

---

☞ In medicina facile est per ea ipsa interdum decipi, quae facere videntur ad vitandas deceptiones.—*Morgagni.*

---

☞ Aegri persuasio et fiducia omni arti et consilio et medicinæ, præferenda.—*Avicenna.*

---

### ADJOURNMENT OF THE JUNE MEETING OF THE AMERICAN MEDICAL ASSOCIATION

### FOR ONE YEAR.—OFFICIAL NOTICE.

---

On a previous page will be found the sentiments of this Journal with reference to this common sense movement. The form containing that article had passed through the press and was "past praying for," a week before the reception of the official notice published below. It is to be expected that some grumbling will be heard from divers and sundry individuals whose only hope of notice in the professional world is their annual exhibition of themselves in the Association, but there can be no doubt that among all sensible, thinking and right-minded members of the Association, this motion will be hailed as one eminently "fit to be made." Reserve your ammunition, Gentlemen of the pop gun order, one year longer, "until you see the whites of the eyes" of your subjects. Bottle up and condense your gas to some degree of sensible solidity. Employ the year in solid work and come next year prepared for some other business than rising to points of order, squabbling over the code, and ventilating your little stock of nonsense about medical education. [*Confidentially.*—The noisiest babblers about raising the standard of medical education we have ever listened to on the floor of the Association, have been those who most astonishingly illustrate in their own persons the necessity for a home-stock of the article.]

The supply of educated medical men will always equal the demand. The force-pump wont improve the matter.

---

MESSRS. EDITORS OF THE CHICAGO MED. JOURNAL:—Please insert the following notice in the May number of your Journal, and oblige your readers. N. S. DAVIS.

*American Medical Association.*—In accordance with the unanimous advice of prominent members of the Association in the Eastern, Middle, Southern and Western States, we, the undersigned Committee of Arrangements, hereby give notice that the Annual Meeting of the American Medical Association, appointed to be held in the city of Chicago, on the first Tuesday in June, 1861, is *postponed* until the *first Tuesday in June*, 1862.

Chicago, May 1st, 1861.

N. S. DAVIS,
J. W. FREER,
E. ANDREWS,
DELASKIE MILLER,
THOS. BEVAN,
J. BLOODGOOD,
H. W. JONES,
} Com. of Arrangements.

### TABLE OF MORTALITY.

### PER CENTAGE IN SEVERAL OF THE LARGER CITIES OF THE U. S. FOR SEVERAL YEARS PAST.

The large comparative mortality in 1854, it will be recollected, depended upon the Asiatic cholera especially cutting down emigrants. The general result of the table scarcely needs comment. Chicago is a healthy city.

| | Boston. | N. York. | Philadel. | Balt. | Charles'n. | N. Orl's. | St. Louis. | Cinn. | Chicago. |
|---|---|---|---|---|---|---|---|---|---|
| 1841— | .... | .... | .... | .... | .... | .... | .... | ..... | .... |
| 2— | .... | .... | .... | .... | .... | .... | .... | .... | .... |
| 3— | .... | .... | .... | .... | .... | .... | .... | ..... | 1.56 |
| 4— | .... | .... | .... | .... | .... | .... | .... | ..... | 2.56 |
| 5— | .... | .... | .... | .... | .... | .... | .... | ..... | 2.38 |
| 6— | 2.59 | 2.61 | 1.70 | 2.35 | 2.09 | 4.13 | 3.80 | ..... | 2.30 |
| 7— | 3.10 | 3.46 | 1.89 | 2.58 | 1.82 | 8.31 | 4.62 | ..... | 2.88 |
| 8— | 2.84 | 3.25 | 1.92 | 2.76 | 2.32 | 6.95 | 3.86 | ..... | 2.60 |
| 9— | 3.79 | 4.64 | 2.28 | 2.84 | 2.75 | 8.05 | 10.62 | ..... | 5.30 |
| 1850— | 2.64 | 3.07 | 1.96 | 2.49 | 2.85 | 6.03 | 5.04 | ..... | 4.72 |
| 1— | 2.68 | 4.05 | 1.97 | 2.40 | 2.25 | 5.25 | 4.39 | ..... | 2.56 |
| 2— | 2.52 | 3.63 | 2.47 | 2.79 | 3.59 | 5.88 | 4.44 | ..... | 3.44 |
| 3— | 2.80 | 3.64 | 2.01 | 2.58 | 2.39 | 10.24 | 3.32 | ..... | 1.99 |
| 4— | 2.82 | 4.46 | 2.39 | 2.89 | 4.27 | 6.57 | 5.35 | ..... | 5.39 |
| 5— | 2.54 | 3.43 | 2.03 | 2.65 | 2.18 | .... | .... | ..... | 2.46 |
| 6— | 2.59 | 3.06 | 2.40 | 2.67 | 2.86 | .... | .... | ..... | 2.17 |
| 7— | 2.36 | 3.16 | 2.09 | 2.55 | 2.36 | .... | 2.88 | ..... | 2.17 |
| 8— | 2.25 | 3.06 | 2.00 | 2.64 | .... | .... | 2.92 | ..... | 2.04 |
| 9— | 2.14 | 2.82 | 1.77 | 2.23 | .... | .... | 3.05 | ..... | 1.75 |
| 1860— | 2.47 | 2 79 | 2.04 | 2.27 | .... | .... | 3.55 | ..... | 1.88 |

*To Subscribers.*—All subscriptions are deemed payable in advance. If any do not happen to be paid thus, we do not in all cases immediately erase the names from the mail book, because the failure, in the large proportion of cases, is merely from oversight on the part of the subscriber. We cannot afford to send out collectors, and therefore hereby constitute each subscriber who is in arrears an agent for the collection of the amount due from himself, and confer upon him plenary powers to send on the *needful* by the earliest ensuing mail after the reception of this notice. As commission on the agency we will insure him prompt transmission of the Journal, and our best efforts to make it readable when received.

*Stricture of the Œsophagus.*—The Journal is promised the details of a case of stricture of the lower portion of the Œsophagus (including the *post mortem* appearances) which had been mistaken by very competent medical observers for cancer in the stomach. The case is one of much interest.

---

*Vaccination.*—The collection of large numbers of men together in camps and barracks suggests the precaution that each should present satisfactory evidences of recent successful vaccination, or immediately be subjected to this important little operation. Small pox in an unprotected camp is a thousand fold more to be dreaded than the bullets of an enemy. Will our army surgeons see to this?

---

*Camp Diseases.*—Germane to the matter just alluded to, we trust that our army surgeons will recollect that in time of war vastly a greater number perish from disease than from "villainous saltpetre" or the sword. Rub up your ideas of dysentery, "malarious" fevers, typhoid, pneumonia, &c., patriotic Brethren; adopt the almost infinitely improved methods of modern practice, and let us hear no more of the awful mortality which has formerly attended these diseases in camp.

---

*Cimicifuga in Epilepsy and Chorea.*—A recent discussion in the Philadelphia Co. Med. Society incidently brought up the use of Cimicifuga in Chorea. Our own experience in this matter has happened to be, perhaps, unusually large. We suggest that the Cimicifuga is useful in both Epilepsy and Chorea occurring at or about puberty, from the ages of 11 or 12 to 18 to 20. It is comparatively worthless either before or after these periods. During this time it controls these diseases with an efficiency unequalled by any other remedy with which we are acquainted. The larger proportion of cases occurring at this age, whether in the male or female, are evidently connected with derangement of the sexual system, and this particular derangement is controllable remarkably by the Black Cohosh. It ought to be added that the Decoction is worthless for this object, the Fl. Extract unreliable, the Powder too bulky and often nauseous. The best form is the saturated tincture of the fresh root. Of this the medium dose is a fluid drachm three or four times a day, continued until a sensation of tightness, or of a band drawn closely over the eyebrows, is produced, when the dose or frequency may be lessened. Of course when this or any other empirical remedy is employed, appropriate regimen and such other medical treatment as may be indicated, should be made use of. *On the whole*, it is a remedy well worth trying in the kind of cases specified—and in these only with any degree of confidence.

---

*Medical Appointment.*—Dr. W. V. Keating ["One of the family"—*Devil.*] has been appointed to the position formerly occupied by Prof. Meigs in the Jefferson Medical College. Pending the appointment there has been given a capital opportunity for the friends of a large number of second and third-rate gentlemen to advertise themselves throughout the country as among "the candidates for the succession." *Facilis decensus, &c.* Be comforted, Gentlemen, remembering that even by Dame Nature you *have* been elected—*to fill a vacancy.*

---

*O si sic omnia!*—The authorities of Aix-la-Chapelle have prohibited all advertisements, either in newspapers or by placards, or otherwise circulated, of therapeutical substances as remedies for diseases of man or beast, under a heavy penalty.

---

SUMMARY OF MEDICAL SCIENCE.—*Edited by Walter S. Wells, M. D.* Part 1, Chas T. Evans 532 Broadway, N. Y. City.

This initial No. of Dr. Wells' Summary fully sustains the high anticipations we were led to express after looking over the same Editor's Epitome of Braithwaite. The typographical execution is of the same character, and the general arrangement and selections, very judicious. It is to be issued semi-annually; single Parts $1.25 or $2.00 per annum in advance.

AMERICAN MEDICAL BIOGRAPHY.—*Edited by Samuel D. Gross, M. D., Prof. Surgery in the Jefferson Med. College of Philadelphia. Lindsay & Blakiston*, 1861.

This work is intended to meet a want felt by every physician who takes interest in the history and progress of the profession.

Since Dr. Thatcher's work, published some thirty years since, and Dr. Williams compilation of sketches issued in 1845, we have been furnished nothing in book form in the shape of medical biography. The larger proportion of the memoirs in the present work have been furnished by various writers, only a few being from the pen of the editor. Most of these are very readable, but we trust in subsequent editions the Editor will depend more upon his own polished pen and singular felicity of style.

We miss many names worthy of niches in this memorial, but understand that the editor intends, should the present volume meet with professional encouragement, soon to send out another which will suitably commemorate those other and noble names, which we look back upon with pride and veneration. The work is to be had at the bookstores for $3.50.

*Per Sulphate of Iron in Post Partum Hæmorrhage.*—Dr. Geo. Mendenhall, of Cincinnati, reports another case of *post partum* hæmorrhage which, not being controllable by ordinary treatment was promptly relieved by intra-uterine injection of two ounces of the saturated solution of per-sulphate of iron. It should be so applied as to entirely wash the inner surface of the uterus. In those rare cases where the usual methods fail, there can be little doubt that this will furnish a most efficient resource.

*Castration for Epilepsy.*—Dr. Jas. J. Rooker, of Castleton, Indiana, reports a case in the *Cleve. Med. Gaz.*, of castration for the cure of Epilepsy evidently dependent upon the habit of excessive masturbation. The patient was thirty-five years of age, and the disease remarkably severe. The operation has proved entirely successful.

---

*Action of Bismuth.*—Prof. J. B. McCaw, in the *Maryland Journal*, gives Bismuth in much larger doses than those usually ordered—sometimes even as much as an ounce in the twenty-four hours. Thus in serous diarrhoeas and "irritable" bowels. So also to neutrallize sulphuretted hydrogen evolved in the intestines. He uses it also as a direct application in erysipelas, burns, excoriations, &c.; also as a general disenfectant in fœtid ulcer, sinuses and malignant sores.

---

*Transactions Am. Med. Association.*—The *Maryland Journal* is evidently much dissatisfied with the results achieved by the late American Med. Association. It considers the seven papers published with the following running commentary. Thus of the Reports on Topography of Drs. Smith and Dickson: "Dr. Dickson's is by far the more practical. Dr. Smith has adopted a more extensive plan, but his details are meagre and there is want of unity between the different portions of his essay." Dr. Pallen's essay, in which he tells us he considers only 'strictly ocular' subjects, seems to us a rather carelessly written compilation."

"As to Dr. McDowell's paper, we are surprised at his having written it, and still more, that it should have been published by the Association. It is simply a loose, sketchy, rambling squib, the burden of which is that the Americans are great surgeons, and need no instructions from Europeans or their books."

"We are glad to be able to speak more favorably of Dr. Sayre's contribution."

"Of Dr. Davis' paper we have nothing to say, because it seems to us to contain absolutely nothing."

"Dr. Ayres has rendered a most important service to a truly beneficent movement."

Dr. McDermont's Report "seems to treat more of a matter of policy than of pure science."

"Dr. Miller's address, although sound in its tendency, touches upon this matter (medical education) in general terms, and does not seem to us to strike at the root of existing evils."

"Nor do we think that the Committee's Report, with respect be it said, covers the whole ground of the difficulty."

Now as these several papers constitute the entire product of the Association from May, 1859, till June, 1860, it *seems* a good deal "*like*" our worthy confrere may have an obscure idea, unexpressed, but very expressible, that the American Medical Association has already "survived the period of its usefulness," and its "mission" being thus far accomplished, our glorious profession may struggle throughout another year unilluminated by its annual volume.

---

*Fistula in Ano.*—The courtiers of Louis XIV. affected a disease of being cut for fistula, in order to commend themselves to the monarch, who was himself unfortunately the subject of the same disease! According to Dionis, many of these personages were greatly annoyed when told that it was unnecessary in their case. "This disease," says Mad. de Sevigne, "was called the '*Maladie du Roi*,' and became the fashion. It was considered *bon ton* to be affected with it; every one wished to be operated on, and many were so, without cause. Dangeau was too prudent a courtier not to conform to the prevailing fashion. He also had the *maladie du Roi*, and submitted to an operation." [In these days the fashion is to have either Diphtheria or, among the sex, ulceration of the womb.]

According to the Journal of the Marquis of Dangeau, it was on Monday, November 18, 1687, at seven o'clock in the morning that Louis XIV. was operated upon. Dionis informs us that he recompensed like a king all his medical attendants. He gave to Felix, his first surgeon, who performed the operation, 50,000 crowns; to Daquin, 100,000 livres; to Fagon, 80,000 livres; to Bessieres, 40,000 livres; four apothecaries had each 12,000 livres, and to Rage, a pupil of Felix, he gave 400 pistoles. Never was the cure of so trifling a disease, and so easy an operation, so magnificently rewarded.—*Ribes.*

